# Enhancing the Therapeutic Delivery of Oligonucleotides by Chemical Modification and Nanoparticle Encapsulation

**DOI:** 10.3390/molecules22101724

**Published:** 2017-10-13

**Authors:** Yating Sun, Yarong Zhao, Xiuting Zhao, Robert J. Lee, Lesheng Teng, Chenguang Zhou

**Affiliations:** 1 School of Life Sciences, Jilin University, Changchun 130012, China; 18744026372@sina.cn (Y.S.); zhaoyr16@mails.jlu.edu.cn (Y.Z.); xtzhao16@mails.jlu.edu.cn (X.Z.); lee.1339@osu.edu (R.J.L.); 2 College of Pharmacy, The Ohio State University, Columbus, OH 43210, USA; 3 Tavistock Life Sciences, San Diego, CA 92130, USA

**Keywords:** oligonucleotide, siRNA, miRNA, antisense, nanocarriers

## Abstract

Oligonucleotide (ON) drugs, including small interfering RNA (siRNA), microRNA (miRNA) and antisense oligonucleotides, are promising therapeutic agents. However, their low membrane permeability and sensitivity to nucleases present challenges to in vivo delivery. Chemical modifications of the ON offer a potential solution to improve the stability and efficacy of ON drugs. Combined with nanoparticle encapsulation, delivery at the site of action and gene silencing activity of chemically modified ON drugs can be further enhanced. In the present review, several types of ON drugs, selection of chemical modification, and nanoparticle-based delivery systems to deliver these ON drugs are discussed.

## 1. Introduction

Oligonucleotides (ONs) targeting RNA have promising therapeutic applications in the treatment of human diseases. However, unmodified oligonucleotides are easily degraded by nucleases and have low membrane permeability due to their high molecular weight and negative charges. Hence, an efficient drug delivery system is needed to improve their therapeutic efficacy [1].

A number of ON-based drugs have received regulatory approval in recent years (Table 1). Vitravene^TM^ was approved by the Food and Drug Administration (FDA) in 1998 [2]. Since then, multiple ON drugs followed [3]. For example, in 2004, Macugen^TM^ (pegaptanib) was approved for age-related macular degeneration [4]. Kynamro™, the first systemic administered ON drug, was approved by the FDA in 2013 [5]. Three additional ON drugs were approved by the FDA in 2016: Exondys 51™ (eteplirsen) was introduced to treat Duchenne muscular dystrophy, Spinraza™ (nusinersen) was approved for spinal muscular atrophy and defibrotide was approved for severe hepatic veno-occlusive disease (sVOD) occurring after high dose chemotherapy and autologous bone marrow transplantation [2]. In 2017, Novartis received FDA approval for CAR T-cell therapy, which is the first gene therapy based on ex-vivo reprogramming of T-cells using a lentiviral vector [6]. Therefore, nucleic acids can potentially be used to polarize the phenotypes’ immune cells and create new therapeutic strategies.

ON therapeutic drugs can be divided into two categories: (1) double-stranded RNAs, including miRNA mimics and siRNA. They usually can be exploited in down-regulating expression of target genes; (2) single-stranded DNA/RNA sequences such as antisense ONs [7]. ONs have been investigated as therapeutic agents for serious diseases such as cancer, genetic disease and acquired immune deficiency syndrome. However, there are several impediments for the clinical translation of ONs. Due to their sensitivity to nuclease degradation, chemical modification of ONs is often necessary to extend their plasma half-life [8,9]. Further improvements can potentially be obtained through encapsulation by nanoparticles [10,11]. Nanoparticles for ON delivery can be based on lipids [12,13], positively charged polymers [14,15], metal nanomaterials and other kinds of natural and synthetic polymers [16,17,18]. To improve the delivery efficiency of nanocarriers, targeting ligands and cell penetrating peptides (CCPs) can be attached to the nanoparticle, or directly linked to the ON in order to enhance the accumulation of the ON drugs at the target site [19,20,21]. In addition, combining ON drugs with chemotherapeutics in nanoparticle-based drug delivery system is a promising strategy that warrant further investigation.

## 2. Mechanism of Action and Major Application of ON Drugs

Understanding the mechanism of action about ONs is important for designing efficient drug delivery system. Although they all work through base complementary pairing, the mechanisms of gene expression inhibition by ONs are different.

### 2.1. siRNA

siRNA is a double-stranded ON with 21–23 bases, which has the advantage of better in vivo stability compared to the single-stranded ONs. Like miRNAs, siRNAs regulate gene expression through RNA interference (RNAi) RNAi was first introduced in 1998 by Fire, Mello and their colleagues [22], for which they were awarded the Nobel Prize. Since then, the number of siRNA related publications have grown exponentially [23].

**Figure 1:**
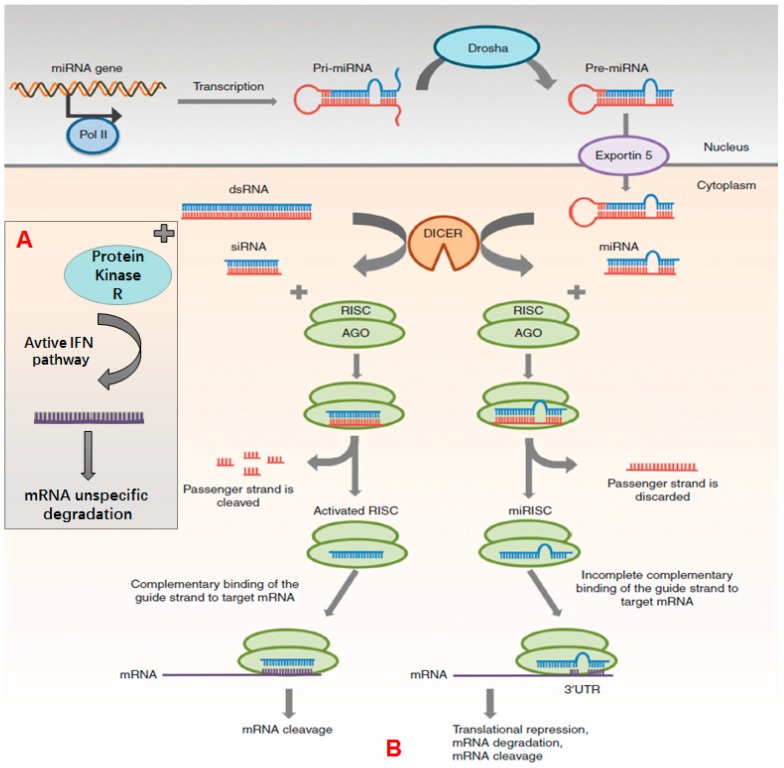
Mechanisms of gene silencing of miRNA, dsRNA and siRNA. Part **A** is the mechanism of dsRNA, and Part **B** is mechanism of RNA interference. Reprinted from reference [24] with permission.

As shown in Figure 1 [24], siRNA is generated from long double-stranded RNA (dsRNA) by Dicer, a specialized ribonuclease (RNase) III-like enzyme that functions in cytoplasm. After processing by Dicer, siRNA is incorporated into RNA induced silencing complex (RISC). Then endonuclease argonaute2 (Ago2) cleaves the sense strand. The remaining antisense strand associates with and activates the RISC to combine with mRNA, causing gene silencing. As the binding of antisense strand with mRNA is based on fully complementary base pairing, gene silencing by siRNA is highly specific.

**Figure 2:**
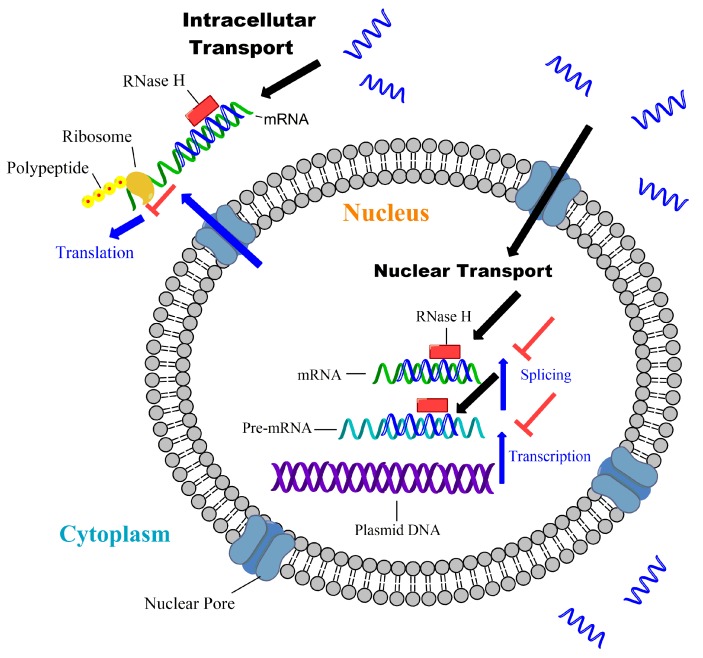
Mechanism of gene silencing by antisense ON. Adapted from reference [7].

Besides siRNA, long dsRNAs (over 30 nucleotides) have recently gained attention. According to Gantier et al. [25], long dsRNA is a key component of the interferon pathway, which takes effect by binding and activating protein kinase R, which in turn activates other pathways of interferon, causing nonspecific degradation of mRNA and cell apoptosis [25] (Figure 2). However, when dsRNAs are developed as therapeutics drugs their immunological properties must be considered. siRNA can also activate the immune system non-specifically.

siRNA-based therapeutic drugs have many applications in the treatment of cancer, acquired immunodeficiency disease, and hereditary diseases. Han et al. prepared nanoparticles which were constructed with galactose-modified trimethylchitosan-cysteine, and loaded with siRNA that inhibit the expression of vascular endothelial growth factor. The nanoparticles accumulated at the tumor site and efficiently inhibited cell proliferation and tumor growth [26]. Intravenous injection is used for siRNA-based drug delivery systems due to their poor oral bioavailabiltiy [27,28]. Apart from cancer treatment, Yu-Wai-Man and her colleagues firstly prepared receptor mediated liposomes loaded with siRNA targeting myocardin-related transcription factor. The siRNA successfully reduced scarring and fibrosis [29]. Dahlman et al. utilized siRNA to normalize the function of endothelium and achieved favorable results in a model of emphysema [27]. Rassu et al. showed BACE1 siRNA and rabies virus glycoprotein co-loaded into nanoparticles showed therapeutic effects in Alzheimer’s disease [30].

### 2.2. miRNA

miRNA was first discovered in 1993 during studies on developmental regulatory genes in *C. elegans* [31]. miRNA is also a double-stranded of endogenous small RNA molecule that negatively regulates gene expression in a post-transcriptional manner. miRNA is produced by several steps (Figure 2). Firstly, miRNA transcript is synthesized by RNA polymerase II to produce primary miRNA (pri-miRNA), with double-stranded stem-loop structure in the nucleus. Then pri-miRNA is processed by a complex of Drosha and DiGeorge syndrome region gene 8 protein (DCGR8) to generate precursor miRNA (pre-miRNA) with 70–100 nucleotides. Then pre-miRNA is transported from the nucleus to the cytoplasm by Exportin 5 and processed by Dicer to generate the miRNA with double stranded ON molecules. Like siRNA, the sense strand is digested by Ago2 of the RISC and the mature miRNA is generated with 18–25 nucleotides. The remaining strand combines and activates RISC, forming miRISC and binding to mRNA through partial complementary base pairing. Hence the regulation and inhibition of miRNA is less specific compared with siRNA. Therefore, a single miRNA can regulate the expression of multiple target mRNAs [24].

According to miRbase statistics, 60% of human protein-coding genes have at least one binding site for miRNA that regulate its expression [32]. Hence, miRNA has multiple applications in cancer [33], and neurodegenerative disorders such as Alzheimer’s disease [34]. Meanwhile, various diseases are found to be connected to miRNA dysregulation. miRNA-based therapeutics can be divided into two approaches: miRNA inhibition and miRNA replacement [24].

For the miRNA inhibition approach, an antisense ON is used [35]. For instance, miR-21, which is highly expressed at tumor cells and promotes tumor growth by inhibiting the expression of dimethylarginine dimethylaminohydrolase 1 (DDAH1), phosphatase and tension homology deleted on chromosome ten (PTEN), and programmed cell death protein 4 (PDCD4) [36,37,38]. In miRNA replacement therapy, synthetic miRNA mimics are used to restore activity of under expressed miRNA. This leads to the inhibition or degradation of target mRNAs. For example, miR-155 plays an important role in promoting cancer cell apoptosis and was found in tumor-associated macrophages (TAM). Liu et al. [33] designed a dual-responsive polypeptide nanocarrier that efficiently transported miR-155 to tumor-associated macrophages (TAM) and repolarized immunosuppressive TAMs to anti-tumor M1 macrophages, promoting the apoptosis of tumor cells. Activation of the immune system is showing great promise for cancer and immune deficiency-related diseases. Kymriah has recently been approved by the FDA as a novel immunotherapy. In this therapy, T-cells are reprogrammed via lentiviral gene and then re-administered to target the tumor [6]. In addition to therapy, miRNA also has application in disease prediction and serves as a biomarker of detection with high sensitivity [39,40]. For example, Chen et al. showed that the expression level of miR-181 family had a strong correlation with brain injury and was found that miR-181c exacerbated brain injury in acute ischemic stroke while other miR-181 families were downregulated [41].

### 2.3. Anitsense ON and CpG ON

Different from miRNA or siRNA, antisense and CpG ONs are—single ON sequences usually comprise 16–21 nucleotides. They can also interfere the expression level of cellular protein by complementary base pairing, and degrade target RNA by RNase H or interfere the metabolism of RNA intermediate through splicing, etc. [3].

ONs can be divided based on mechanisms of action (Table 2), e.g., antisense ON, splice switching, CpG-containing ON and triple-helix-forming ON [42]. As for antisense ON, it usually inhibits the target mRNA, particular DNA sequence or its promoter [43]. After binding to the target nucleic acid sequence, antisense ON can hybridize to the mRNA to stop its binding to the ribosome. When the antisense ON is coupled with RNase H, the target nucleic acid sequence is digested by the active RNase H. As shown in Figure 2, there are mechanisms of actions for antisense ONs J [43]. Splice switching ON plays an important role in RNA repairing and modulation, through inhibiting or promoting exon insertion to modify the splicing pattern of pre-mRNA [44,45]. CpG-containing ON is a sequence that composes numerous unmethylated CG dinucleotide. CpG ON can trigger cells to express toll like receptor 9, and induces innate immune response through producing Th1 and pro-inflammatory cytokines. Based on this mechanism, CpG ON can be used as vaccine adjuvants and act by improving antigen presentation and generating specific cellular and humoral responses. Bai et al. used CpG ON as vaccine additive to treat Hepatitis B, due to its ability to promote binding capacity of naïve B cells to the Hepatitis B virus epitopes [46]; Triple-helix-forming ON is formed by single strand ON inserting into the double stranded DNA. After the insertion, double stranded DNA can’t transcribe mRNA and therefore relevant protein expression is inhibited [47].

## 3. Barriers and Limitations for Systemic ON Drugs Delivery

**Figure 3:**
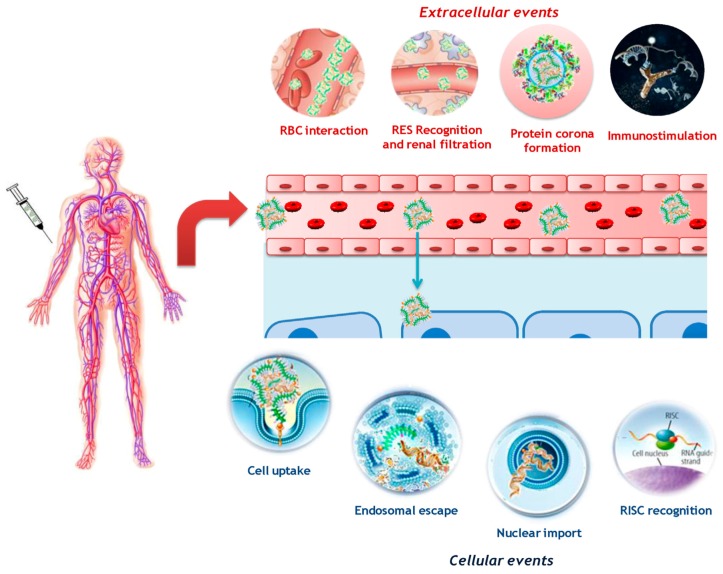
Barriers to ON drugs loaded into nanocarriers. Reprinted from reference [14].

Although ON sequences have multiple applications in cancer, acquired immune disease, congenital genetic diseases and vaccines, there are several barriers to ON delivery in vivo (Figure 3). Naked ONs are easily degraded by the nucleases in plasma, and the half-life of naked ONs is about 5 min according to pharmacokinetic studies in primates [48]. To extend the biological half-life of ONs, several strategies were taken such as chemical structure modification in 2′-ribose sugar or phosphate groups, which increased the plasma half-life to 30–60 min according to the research of Geary et al. and Zhang et al. [49,50]. Besides chemical modification, application of nanoparticle-based delivery systems also can solve the problem of short biological half-life. Nanoparticles, liposomes and micelles have been utilized to deliver ONs and shown to successfully prolong their plasma half-life [14,51,52]. Combining chemical modifications and nanoparticle-based delivery is the most promising approach for therapeutic ON delivery.

There are multiple obstacles to deliver ONs to the target site, mainly divided into three levels, organ level, tissue level and cell level. A major issue is uptake by the reticuloendothelial system (RES). When nanocarriers loaded with ONs are injected into blood vessels through an intravenous route, multiple serum proteins will absorb onto the surface of nanocarriers, hence promoting clearance by the mononuclear phagocyte system (MPS). Through opsonization, nanoparticles loaded with nucleic acid drugs accumulate in the liver and spleen, which may cause side effects [53]. Therefore, PEGylation is typically used to reduce protein adsorption to the surface of the nanoparticles [54]. Meanwhile, serum albumin has been used to modify the nanocarriers, and successfully resolve the aforementioned issues [55]. Besides, targeting ligand modification of nanocarriers can enhance a drug’s accumulation at the target site [56]. However, the shearing stress in flowing blood may destroy some nanoparticles, then ONs are released and degraded by nucleases [53]. After escaping from RES, nanocarriers loaded with ON will have to extravasate from the endothelium of the blood vessels to the target cells. Among various cells, cancer cells are quite difficult to get close due to the tight extracellular matrix and high pressure of tumor tissue. When the nanocarriers passively diffuse to the periphery of tumor cells, there are still intracellular barriers that need to be overcome. As nanocarriers are too large to access cancer cells through passive diffusion, this mainly occurs by endocytosis or pinocytosis. When the nanocarriers are contained in the endosome, there is still an intracellular barrier for nanocarriers loaded with ONs to successfully escape from the endosome and release ONs into the cytoplasm. Besides the ONs still need to penetrate the nuclear envelope to inhibit gene transcription when ON works on a DNA transcriptional level. It should be noted that the nuclear envelope is absent during cell mitosis, which enables nuclear delivery into dividing cells [7]. In general, through all the barriers, majority of nanoparticles are eliminated and only a few nanoparticles can reach tumor sites when administered for cancer treatment.

## 4. Common Chemical Modification Strategies for ON Drugs

Cell membranes consist of lipid layers. Negatively charged ONs cannot pass through the cell membrane. Besides, humans have developed a variety of mechanisms to prevent infection by pathogens during the long evolutionary processes, which form barriers to unmodified ONs. Therefore, strategies are needed to improve the stability of ON drugs and the cellular uptake rate to achieve the desired therapeutic effects. One is chemical modification of the ON molecular structure, the other is the use of targeting ligands or cell penetrating peptides (CCPs) to conjugate ONs. The ligation product can then be loaded into nanocarriers to further facilitate transport.

### 4.1. Structural Variants of ONs

ONs have great potential in the treatment of several diseases, but lack stability in physiological fluids and have poor cell penetration ability. These severely limit the clinical application of ONs [57]. To enhance the stability of ONs, chemical modifications are needed, including the modification of ON skeleton on the phosphodiester bond, the modification of ribose, base modification, and the use of ON analogues to replace ON skeleton (Table 3) [58]. A number of chemical modifications have significantly enhanced their metabolic stability and their affinity for RNA targets, and have reduced the off-target effect to a certain extent [59].

#### 4.1.1. The Modification of the Diester on the ON Skeleton

Thiophosphate modification is a common tactic used in the chemical modification of ONs [58]. One of the non-bridge oxygen atoms in the diester bond is replaced by sulfur. Chemical modification can help the body enhance cellular uptake and increase the bioavailability of the modified ONs. In the meantime, the resistance to circumscribed nucleases is also effectively increased; so most therapeutic ONs and several double-stranded RNA are modified more or less recently [25,62]. However, although the modified siRNA is found to be significantly stable in the body, it increases the cytotoxicity and decreases the gene silencing effect [63]. Several studies have shown that the modification of phosphorylated phosphate ester in the phosphorylation location damages RISC activity [64], and it is not recommended to modify the siRNA using thiophosphate.

#### 4.1.2. Modification of Ribose

Because the gene silencing activity of siRNA or miRNA does not depend on the 2′-OH groups of ONs, researchers have often used other chemical groups to replace this group for the modification of the structure [24]. The common strategies are 2′-O-methylation, 2′-oxygen-allylation and 2′-fluorization modification, which can enhance the stability of the double strand. When the ONs are modified by 2′-O-methyl and 2′-methoxyethyl, they no longer support the enzyme H to degrade mRNA, reducing the overall gene silencing effect of antisense ONs. Studies have shown that certain allylation modifications might reduce the activity of the siRNA. The widespread use of thiophosphate modifications results in a certain cytotoxicity, but the 2′-O-methylation improves the siRNA activity and is nontoxic to normal cells [60]. The activity of siRNA depends on the position of the modified parts, extensive or complete modification may result in a significant loss of gene silencing, for example, the completely 2′-O-methylated siRNA is inactive [24]. By alternately replacing 2′-O-methyl and 2′-fluoro of the full siRNA, the resistance to nucleases and the gene silencing effect are efficiently enhanced [65].

#### 4.1.3. Base Modification

The modification of the base plays an important role in the function of ONs, for example, it can improve the function of the siRNA and increase the ability of the siRNA interaction with the target mRNA. Meanwhile, the modification increases the ability of RISC to recognize and cleave the mRNA. The chemical reactions of the functional domain in the base sequence mainly include electrostatic interaction, hydrogen bonding, complexation and especially the generalized acid-base interaction. Specifically speaking, the modifications on the base include adenine methylation and deamination, cytosine methylation, hydroxymethylation and carboxy substitution, and guanine oxidation, etc. [61]. The modified bases are all related to the changes of functional groups, which is the basis of triggering the functional changes through the modification of structure of ONs.

#### 4.1.4. ON Analogues Replace the ON Skeleton

Skeletal modifications are commonly used to reduce the degradation of ONs by nucleases through the use of other types of phosphodiester scaffolds, e.g., peptide-substituted, and the resulting materials typically include peptide nucleic acids, locked nucleic acids, and morpholinophosphamides, which have low toxicity and a slight decrease in affinity compared with unmodified sequences [57]. These nucleotide analogs do not support the cleavage of RNase H-mediated target mRNA in antisense ONs, thereby they primarily exhibit their reflective activity by steric hindrance to prevent gene expression during transcription or translation. This method further enhances the binding affinity, nuclease resistance, and more targeting effect compared with several other chemical modifications.

### 4.2. CCPs and Ligands Conjugates

Apart from structural modification of ONs, different CCPs and ligands conjugated to ONs-based drug delivery system are normally adopted following the conjugation. CCPs are a class of short peptides that are rich in cations and can efficiently enter cells through penetrating biofilms. Based on these properties, CPPs are used to modify DNA, RNA and ONs and loaded on nanocarriers for therapy. The conjugation of ONs and CPPs can overcome the deficiencies of cytotoxicity and enhance the efficiency in eukaryotic cells.

Complexes formed by cationic CPPs and anionic ONs which are formed through electrostatic interaction can promote ONs’ entry into cells and initiate RNAi, leading to silencing of endogenous genes [66]. Nanoparticles can potentially further enhance the activity of such complexes. For example, the 5′ end of the siRNA can be modified with the free thiol group of the amino acid cysteine on the CPPs, then the CPPs-siRNA are encapsulated into ultrasound-sensitive nanomicrobubbles (NBs). Jing et al. used this method to prepare CPPs-NBs that loaded siRNA targeting epidermal growth factor receptor (EGFR) to triple negative breast cancer cells. When NBs reached the target site, they were disintegrated under external ultrasonic irradiation, releasing CPPs and siEGFR to achieve cytoplasmic delivery. Since CPPs had a strong nonspecific binding effect with all the cells, it is beneficial to attach a targeting ligand onto the NBs to induce cell specific binding and receptor-mediated endocytosis [67]. Therefore, CPPs-siRNA in NBs has been modified with a targeting ligand and triggered to be delivered by ultrasound [68]. It was shown that ultrasonically sensitive nanoparticles loaded with CPP-siRNA behaved well in gene delivery and would achieve long-term development in cancer gene therapy. Peptide nucleic acids (PNAs), which are neutral, can replace ONs, which are negatively charged, to improve delivery. Chaubey et al. conjugated CPPs and PNA successfully with specific targeting and demonstrated high cellular uptake. Meanwhile, the construct showed little toxicity up to a dose of 300 mg/kg [69].

Transactivator of transcription (TAT) peptide was conjugated to ONs and loaded onto nanoparticles and shown to improve delivery [70,71]. Gelatin has been coupled with ONs to form nanoparticles. To improve the affinity between gelatin molecules and siRNA, the two molecules were chemically modified to form the thiolated gelatin (tGel) and the 5′-terminal sulfhydryl modified siRNA separately, and these two modified components were cross-linked through disulfide bond. Then polymeric siRNA was encapsulated in the self-assembled tGel nanoparticles [72]. In summary, a combination of chemical modification and a nanoparticles-based drug delivery system is likely to be more effective for ON delivery.

## 5. Drug Delivery Systems for ONs

Using ONs to treat diseases will encounter various obstacles, although physical and chemical modification have made up some of their deficiencies. Different nanocarrier strategies are still needed to adopt in practical applications and make them more effective in diagnosing and treating diseases. The requirements of a ON delivery system include biocompatibility, biodegradability and non-immunogenicity. Importantly, the nanocarriers should protect ONs from the adsorption of serum proteins and degradation of nucleases, and effectively deliver them to targeting cells. After entering the cells, the nanocarriers should ensure that the ON can escape from the endosomes and enter the cytoplasm. To solve the problems on the delivery procession of ONs, many nanoparticle-based delivery systems have been developed.

### 5.1. Liposomes

#### 5.1.1. Cationic Liposomes

As mentioned above, ONs are negatively charged and easy to be encapsulated into cationic liposomes through electrostatic adsorption, so cationic liposomes are widely used as ON delivery systems [7]. As the main component of cationic liposomes, cationic lipids consist of a cationic head and a hydrophobic chain, while the cationic head is the main part of the reaction with anionic ONs. Cationic lipids include monovalent lipids and multivalent lipids. As shown in Table 4, monovalent lipids include DODMA [73] and DOTAP [74]. The selection of cationic head and hydrophobic chains may significantly affect the transfection efficiency and toxicity of cationic liposomes [24]. Generally, the transfection efficiency of cationic liposomes containing multivalent lipids is higher than in monovalent lipids.

Cationic lipids interact with ONs by electrostatic complexation and besides the cationic head function, the alkyl chain is also important for the endosomal escape of the liposomes. The length and saturation of alkyl chain are related to the fluidity of liposome membranes. Short chains and unsaturated chains often increase the fluidity of the lipid membrane and sequentially improve the transfection efficiency of ONs [70]. In addition, neutral helper lipids play an important role in promoting the stability and endosomal escape of liposome-encapsulated ONs. DOPE is one of the most widely used neutral helper phospholipids [75].

As mentioned before, PEG is a hydrophilic polymer that can be used to modify nanocarriers. It has the advantages of increasing the duration of nanocarriers in the blood circulation, and partially screening the positive charge in cationic liposomes which can decrease the cytotoxicity and prevent the adsorption of serum proteins. Most of the delivery systems for cationic liposomes-containing ONs have been modified with PEG to achieve the better delivery efficiency in vivo [43].

Besides cancer treatment, there are other diseases that can potentially be treated by ONs. Aiming at solving the problems of complexity and viscoelasticity of the gastric mucus and the unpenetrability of the cell envelope of *Helicobacter pylori* (*H. pylori*), Santos et al. evaluated the characteristics of fusogenic stealth liposomes that were composed of DOTAP-DOPE liposomes and post-PEGylated with DSPE-PEG (DSPE Lpx) delivering nucleic acid mimics (NAMs) to target *H. pylori* [12]. Studies about the delivery of ONs to bacteria through liposomes for disease diagnosis and therapy are rare. This kind of fusogenic stealth liposomes provide the possibility of inhibiting *H. pylori* in gastric mucus.

Remaut et al. compared the efficiency of pegylated liposomes and non-pegylated liposomes as nanocarriers for delivering antisense ONs [76], and the results showed that although pegylation brought benefits for liposomes at the circulatory systemic level, the presence of PEG inhibited endosomal escape, causing ON degradation in endosomes. By comparison, non-pegylated liposomes could efficiently escape from the endosomes and into the cytoplasm of the cells. It is therefore desirable to use PEG anchored to the lipid bilayer with a short hydrophobic anchor, such as PEG-DMG.

#### 5.1.2. Neutral Liposomes

Neutral liposomes are primarily constructed by neutral lipids, which include PC, PE, cholesterol [7] and DOPE (Table 4). DOPE can not only be used as an adjuvant phospholipid in cationic liposomes, but also commonly be used to prepare for nanocarriers for ONs to enhance the transfection efficiency. Neutral liposomes have good biocompatibility and excellent pharmacokinetic characteristics [70], but they can’t interact with ONs to adsorb them and encapsulate them into the liposomes efficiently.

#### 5.1.3. Ionizable Liposomes

Ionizable liposomes are important nanocarriers for siRNA delivery [81]. Unlike the cationic liposomes and neutral liposomes with a single type of charge, ionizable liposomes can be protonated and deprotonated according to the environment acidity. They are ideal liposomes that can carry positive charges when the ONs are loaded into liposomes, and lose their charges after administration but before entering the cells. Then, they can regain positive charges after entering the endosomes for escaping [7]. Therefore, pH-sensitive ionizable liposomes are developed and successfully accommodate the need of delivering ONs. In general, ionizable liposomes are constituted by amino lipids that have ionizable amine headgroups, and the pKa value of the ionizable amino in liposomes is an important factor in considering the carrier design, because the pKa plays an important role in determining the interaction of amino acids with cell membranes and serum proteins, which ultimately determines the delivery effectiveness and toxicity of the liposome [81].

Based on low-pH and hypoxia conditions in tumor microenvironment, novel ionizable liposomes were designed to deliver siRNA into glioma cells by Liu et al. Ionizable liposomes constituted by tertiary amines are the most frequently used [76]. Tertiary amines maintain positive charges in acidic environments and have neutral or small positive charges in blood circulation. Under hypoxia condition, tumor tissues are more acidic, then pH-responsive liposomes have more positive charges. The nitro group of nitroimidazoles can be restored to become amino groups under hypoxic conditions, so liposomes containing nitro moieties can acquire more positive charges under low oxygen conditions, and improve the uptake in tumor cells. Habrant et al. prepared several different ionizable liposomes based on the aminoglycoside tobramycin, which is a cationic agent [83]. The results demonstrated that these liposomes loaded with ONs had high transfection efficiency.

Comparing the three types of liposomes, cationic liposomes are widely used in ON delivery. Meanwhile, the applications of neutral liposomes are relative few, and neutral lipids are adopted to modified cationic liposomes to enhance particle stability. Ionizable liposomes have extensive applications in future development due to their better activity in vivo.

### 5.2. Micelles

Polymeric micelles have promising applications in drug delivery, because they show improved pharmacokinetics and biocompatibility compared to other carriers [84]. Amphiphilic copolymers are typically used for synthesizing polymeric micelles. The common hydrophilic material for polymer micelle synthesis is PEG, while the hydrophobic components mainly include polyamino acids, polylactic or glycolic acid, polycaprolactone and short phospholipid chains, etc. The self-assembly of amphiphilic polymers can improve the stability of colloids and reduce the non-specific interactions between micelles and other biomolecules. Besides, polymer micelles have additional advantages for drug delivery, including extending the drug cycle time, changing the drug release curve and easily connecting targeted ligands.

Cationic polymer micelles have been shown as effective nanocarriers to deliver ONs. The positively charged cations ensure good ONs loading capacity through electrostatic adsorption. Besides, cationic polymer micelles have a nanoscale, unique core/shell structure, long circulation times and tumor passive targeting by the EPR effect. In addition, cationic polymer micelles may have the advantage of efficient ON endosome release by the proton sponge effect [85]. Besides the conventional cationic amphiphiles, novel polymeric micelles have been developed to improve the transfection efficiencies of ONs and the therapeutic effect on diseases. Recently, novel cationic amphiphiles such as polyethylenimine-poly[*ε*-(-caprolactone)-*co*-glycolide]-polyethylenimine (PEI-CG-PEI) and poly (ethyleneglycol)-b-poly(l-lysine)-b-poly(l-leucine) (PEG-PLL-PLLeu) [86,87] have been reported and exhibited good loading capacities for ONs.

Gwak et al. synthesized a new cationic amphiphilic copolymer, poly (lactide-glycolide)-graft-polyethylene imine (PgP). PgP self-assembly formed cationic polymeric micelles connecting connected polylactic (4 kDa) and branched polyethylene imine (bPEI) (25 kDa) at the ratio of 3:1 to deliver ONs to change the gene expression level for the treatment of spinal cord injury. The results showed that PgP provided high level of transgenic expression in the spinal cord of rats, significantly greater than the transgenic expression obtained by traditional bPEI. So PgP may be a promising polymeric micelle for nerve regeneration by ON delivery [88].

As we all know, many drugs are not effective because of the multidrug resistance (MDR) of tumor cells, so overcoming multidrug resistance is especially important in the treatment of the disease. Based on this fact, the combination of RNAi and chemotherapy is a new strategy for treating multidrug resistant diseases. The suitable carrier should can deliver ON and chemotherapy drugs together to the tumor tissue and release the two drugs simultaneously. Jacin et al. reported a self-assembled micelle containing a hydrophilic β-cyclodextrin-polyethylenimine (PEI-CyD) terminal and a cholesterol hydrophobic terminal. Doxorubicin (DOX), a hydrophobic p-glycoprotein substrate, was wrapped in the hydrophobic core. At the same time, siRNA interacted with the external PEI-CyD by electrostatic adsorption, which could downregulate the expression levels of mRNA and P-glycoprotein. This special structure ensured that the sequential release of siRNA and drug. MDR was inhibited by RNAi first, then drug concentration was increased, completing the task of killing cells. In vitro and in vivo studies demonstrated that the polymeric micelles could produce the combination of chemotherapy drugs and ONs to reverse MDR. Meanwhile the two drugs have synergistic effect in inhibiting tumor growth [89].

### 5.3. Nanoparticles

#### 5.3.1. Albumin-Based Nanoparticles

Human serum albumin (HSA) is a plasma protein. Tumor cells can take up HSA through endocytosis. As for ONs, HSA can’t interact with them since HSA has negative charges in aqueous media, thereby, HSA requires chemical modification or the addition of positive charges to introduce charge interactions between HSA and ONs. There are different methods for preparing HSA nanoparticles. Son et al. introduced thiols onto both HSA and siRNA, and the thiolated HSA (tHSA) and the polymeric siRNA (psi) formed a stable nanoparticle (psi-tHSA) by disulfide bond crosslinking. These nanoparticles showed effective gene silencing activity without obvious cytotoxicity [90]. Wen et al. prepared HSA nanoparticles loaded with ONs by physical interaction. HSA has an isoelectric point (pI) was 4.7 at pH 4.0, HSA and ONs formed the nanoparticle via electrostatic interaction, when nanoparticles were heated to 75 °C for 15 min, the structure and particle size of nanoparticles became stable. The data showed that HSA nanoparticles loaded with ONs were stable and had a narrow distribution of about 110 nm in diameter. Experiments with cells demonstrated that the empty nanocarriers showed high cellular uptake and no toxicity to Hela cells [91].

Ming et al. developed a morpholino-ON transporting system constructed by albumin modified with RGD peptide as targeting ligands and then using chemical methods to combine the ONs with RGD peptide, and making connections to the HSA molecule chemically. The results demonstrated a 61-fold enhancement through RGD-mediated cellular delivery to tumor cells when compared with the non-targeted nanocarriers. GD modified nanoparticles also had high gene silencing effects at low concentrations of ONs [55].

Through various methods, HSA nanoparticles loaded with ONs are successfully manufactured and can be modified with targeting ligands. Compared with other nanotechnology-based drug delivery systems, HSA nanoparticles have better stability, because of they have less serum protein interaction. Hence, HSA nanoparticles have wide applications in ON drug delivery considering the reduced opsonization, while other nanodrug delivery systems need PEG modification to reduce serum protein absorption [54].

#### 5.3.2. Metallic Nanoparticles

Since metallic nanoparticles have the property of the enhanced surface to volume ratio compared with macromolecular materials, metallic nanoparticles have good applications in the medical and pharmaceutical fields, e.g., for ON delivery and targeting cancer therapy [92]. In particular, metallic nanometer-sized materials, such as silver, gold, copper and titanium, all show favorable physical and chemical properties, and significant antibacterial activity [93]. Besides, the metallic oxide iron oxide has the property of magnetism forms magnetic nanoparticles as a magnetic material [94].

Gold nanoparticles (AuNPs) have shown great potential as siRNA delivery carriers for the treatment of various malignant tumors. Due to their advantageous properties, such as bio-inertness, easily modifiable surfaces, controllable particle size and shape in the synthesis process, while protecting ONs from RNA enzymes and obviously increasing the circulation time [95,96].

Rahme et al. synthesized some positive, non-surfactant AuNPs though the method of reduction. These AuNPs had good cell activity and the potential in siRNA delivery, but they were unstable in vivo due to the presence of positive charges, therefore, PEG was adopted to improve the stability of the AuNPs in the serum environment. PEGylation shelters part of the positive charge, reducing the interactions with serum proteins to enhance the biocompatibility of the AuNPs and avoiding aggregation, while protecting the ONs from degradation [95]. As an antibacterial agent, silver has the potential to be a nanocarrier for drug delivery. Silver nanoparticles have unique electronic, optical and chemical properties. The particle size and surface coating of silver nanoparticles (AgNPs) play important roles in antimicrobial activity, and smaller silver nanoparticles are observed to be more toxic [93]. Sun et al. prepared AgNPs loaded with quercetin (AgNPs-Qe) and siRNA molecules to enhance the antibacterial activity against drug-resistant bacteria. The results showed that siRNA/AgNPs-Qe could destroy cell walls and inhibited bacterial reproduction. At the same time, after the intravenous injection of siRNA/AgNPs-Qe into mice, the bacteria in the blood, organs and inflammatory cells gradually reduced, while AgNPs and AgNPs-Qe were not as effective as siRNA/AgNPs-Qe in vitro and in vivo [97].

Like gold and silver nanoparticles, magnetic nanoparticles are easy to synthesize and modify [98]. Besides, magnetic nanoparticles can be manipulated by an external magnetic field and realize the targeted delivery. Previously, superparamagnetic iron oxide nanoparticles (SPIONs) were used for siRNA delivery and had limited activity. To solve these problems, a PEG coating was added to enhance the stability and stealth of nanoparticles. In addition, adding the polymer of polyl-l-arginine was found to improve the efficiency of siRNA transfection [99].

#### 5.3.3. Other Common Nanoparticles

There are numerous diverse types of polymer and polymeric hybrid nanoparticles and most of them utilize ligands to modify nanoparticles, which makes them have the ability to target specific cells or tissues, and lead to more effectively delivery.

The load capacity, degradation rate and release kinetics of gelatin nanoparticles are adjustable, but natural gelatin nanoparticles are not useful as siRNA carriers [90]. However, it is easy to introduce specific ligands into gelatin nanoparticles [91] for targeting specific organs [89]. Gelatin can be easily modified with a number of functional groups and has shown low cytotoxicity and antigenicity.

Cationic chitosan can effectively encapsulate ONs and form nanoparticles. Besides, chitosan has good biocompatibility and biodegradability properties, which make chitosan a potential nanocarrier candidate in delivering ONs [72].

The nanocarriers used to deliver ONs are diverse, but they are all characterized by consistent biocompatibility and biodegradability. In addition, as good ON delivery nanocarriers, they should have strong ON loading capacity and efficient transfection capacity, so they are able to overcome some of the obstacles mentioned above to achieve the purpose of healing disease.

### 5.4. Targeting of Nanoparticle-Based Delivery Systems

Cell-specific targeting can be achieved through receptor-mediated endocytosis by conjugating the appropriate ligand to the nanocarriers. Common ligands include antibodies, transferrin, folic acid, RGD peptides, carbohydrates and lipids [63]. Targeting ligand-modified delivery systems can protect ONs from nuclease degradation, and therefore can avoid complex chemical modifications of the ON skeleton. Meanwhile, their site-specific delivery also improves the efficiency of drug delivery [23]. Pegylated nanoparticles conjugated to a targeting ligand are often used for ON delivery for increasing systemic circulation time. Pegylation was shown to increase the stability of the complex for heparin replacement ONs and reduce nuclease degradation [23]. Zhang and his colleagues attached the pH sensitive polyethylene glycol (PEG) chain and membrane peptide to the surface of the liposomes, meanwhile, utilized the PEG chains to cover the CPPs on the surface of the liposomes. The mild acid tumor environment triggered the removal of the PEG chain, thereby activated the CPPs which enabled ON delivery across the cell membranes [64].

Lipid-conjugated polymers can form nanoparticles effective for ON delivery [100]. For example, folic acid conjugated carbamate-choline phosphate copolymer was shown to form colloidal complex with DNA and facilitate cellular transfection [101]. Leukemia cells or human immunodeficiency virus (HIV) infected cells have been successfully targeted using antibody-coupled nanoparticles [102,103,104].

## 6. Conclusions

ONs have shown significant potential as therapeutic agents for various kinds of diseases such as cancer, neurological disease, infectious disease and hereditary diseases. Although there are many barriers for the use of ONs as real medicines in clinical application, progress has been made in recent years. Various modifications of the chemical structure of ONs effectively solve their stability issues in plasma and simultaneously enhance the binding efficiency to the target genes. Besides that, various nanoparticle-based delivery systems have been adopted to encapsulate ONs and make the ONs accumulate more in the target tissue.

Among the disease treatments by nanoparticle-based drug delivery system, tumors are frequently the target. To enhance the accumulation of nanoparticles at the target site and reduce the off-target effects, targeting ligands are usually used to modify the surface of nanocarriers, and CPPs are often utilized to connect ONs and this vastly enhances the nanocarrier delivery system’s ability to penetrate barriers. Through these modifications, the ONs are more accumulated in the tumor site. To magnify the tumor inhibition effect, ON combinations with chemotherapeutic drugs co-loaded into nanocarriers is an emerging strategy in recent years.

## Figures and Tables

**Table 1:** ON-based drugs approved by the FDA.

Trade Name	Time to Market	Company	Indication
Vitravene	1998	Isis (Ionis)	Cytomegalovirus-induced retinitis
Macugen	2004	Pfizer/Eyetech	Age-related macular degeneration
Kynamro	2013	Sanofi/Isis (Ionis)	Familial hypercholesterolemia
Exondys 51	2016	Sarepta Therapeutics	Duchenne muscular dystrophy
Spinraza	2016	Biogen/Ionis	Spinal muscular atrophy
Defibrotide	2016	Jazz Pharma	Severe hepatic veno-occlusive disease

**Table 2:** The mechanism and applications of different ONs.

Category	Mechanism of Action	Mainly Applications	Reference
Antisense	Hybridizes with mRNA and inhibit ribosome’s binding	Cancer treatment congenital genetic disease and acquired immune disease treatment	[43]
ON	Coupled with RNase H and promote targeting nucleic acid’s degradation		
Splice switching ON	Inhibits or promotes exon insertion to modify pre-mRNA’s splicing pattern	RNA repairing and modulation	[44,45]
CpG-ON	Triggers cells to express toll-like receptor 9, and induces innate immune response	Vaccine adjuvants	[46]
Triple-helix-forming ON	Inserts into double stranded DNA to inhibit mRNA transcription	Virus infection treatment cancer treatment	[47]

Annotation: CpG-containing ON is a sequence that composes numerous unmethylated CG dinucleotide. Mechanisms of gene regulation by siRNA, miRNA and antisense ONs are all based on base pairing. The gene inhibition by antisense ONs and siRNA is for a single target. In contrast, a miRNA can regulate many mRNAs. Meanwhile, antisense ONs inhibit mRNA by holding back mRNA binding to ribosome. Meanwhile, long dsRNA and CpG-containing ON can function through activating toll-like receptors. At the same time, splice switching ON takes a role in modifying mRNA’s splicing pattern and triple-helix-forming ON inhibit mRNA transcription by inserting double stranded DNA as shown in Table 2.

**Table 3:** Structural modification of ONs.

Category	Contents	Strength	Shortage	Reference
Diester modification	Phosphorothioate	Increase cellular uptake, bioavailability and resistance to nucleases	Cytotoxicity increases, gene silencing effect decreases	[58,59]
Ribose modification	2′-O-Me, 2′-O-A, 2′-F	Enhanced stability	Gene silencing effect decreases	[24,60]
Base modification	Adenine methylation and deamination. cytosine methylation, hydroxy methylation and carboxy substitution, Guanine oxidation	Improved gene silencing effect	Functional groups change easily through modification	[61]
ON analogues replacement	Peptide nucleic acid, locked nucleic acid, morpholino phosphamide	Good targeting effect, nuclease resistance	Binding affinity decreases	[57]

**Table 4:** Various kinds of LPs and their description.

Groups	Materials	Strength	Limitations	Normal Method	Reference
Cationic LPs	DOTAP	Positive charge, high encapsulation efficiency, easily access to cells, endosomal escape	Adsorption of anionic serum proteins, fast clearance by RES	Neutral lipid and pegylated modification	[73,74,77,78]
	DODMA				
	DOGS				
	DC-Chol				
Neutral LPs	PC	Good biocompatibility and pharmacokinetic characteristics	Low encapsulation efficiency	Adding cationic materials	[7,75,79,80]
	Chol				
	DOPE				
Ionizable LPs	DODMA	Transformable charge, high transfection efficiency, broad prospects		Improve design ideas	[7,81,82,83]
	DODAP				

## Abbreviations

2′-O-Me	2′-O-methylation
2′-O-A	2′-O-allylation
2′-F	2′-fluorization
DODMA	1,2-dioleyloxy-*N*,*N*-dimethyl-3-aminopropane
DOTAP	1,2-dioleyl-3-trimethylammonium propane
DOGS	dioctadecylamidoglycylspermine
DC-Chol	3β-(*N*-(*N*′,*N*′-dimethylaminoethane)-carbamoyl) cholesterol
PC	phosphatidylcholine
PE	phosphatidylethanolamine
Chol	cholesterol
DOPE	1,2-dioleoyl-sn-glycero-3-phosphoethanolamine
DODMA	1,2-dioleyloxy-*N*,*N*-dimethyl-3-aminopropane
DOTAP	1,2-dioleyl-3-trimethylammonium propane
DOGS	dioctadecylamidoglycylspermine
